# Post-surgical Hypertrophic Scar in a Patient With Unilateral Temporomandibular Joint Ankylosis

**DOI:** 10.7759/cureus.67344

**Published:** 2024-08-20

**Authors:** Prasad Cheruvathur, Triveni Palani, Arun Kumar Kamalakaran, Taranitha Krishnamoorthy, Lavanya Lakshminarasimhan

**Affiliations:** 1 Department of Oral and Maxillofacial Surgery, Tamil Nadu Government Dental College and Hospital, Chennai, IND

**Keywords:** temporomandibular joint, wound healing, al-kayat bramley incision, tmj ankylosis, hypertrophic scar

## Abstract

Wound healing is nature’s response to injury. It is a complex and dynamic process involving multiple biological systems aimed at restoring the integrity of damaged tissue. The temporomandibular joint (TMJ) is a critical anatomical structure that facilitates jaw movement and function. TMJ ankylosis is a pathological condition characterized by fusion of the mandibular condyle to the glenoid fossa resulting in severe restriction in mouth opening and significantly reduced mandibular movements. This condition affects the patient’s quality of life by deteriorating major functions such as mastication, speech, oral hygiene, breathing, facial growth, and esthetics. Gap arthroplasty is the mainstay of treatment. There are various surgical approaches to TMJ such as Al-Kayat Bramley, Popowich’s modification, Blair’s inverted hockey stick, Dingman’s, Thoma’s, endaural, postauricular, and rhytidectomy incisions. Wound healing in the TMJ region poses unique challenges due to its complex anatomy and the high level of mechanical stress it endures. Following trauma to TMJ, hematomas are organized by fibrous granulation tissues and mesenchymal stem cells are recruited from adjacent bone by cytokines and chemokines such as bone morphogenetic proteins, transforming growth factor-beta and stromal cell-derived factor 1. These recruited mesenchymal cells differentiate into osteoprogenitors and osteoblasts to form new bone and fibroblasts to form a scar. In humans, scarring is the final outcome of the wound healing process, which has evolved to rapidly repair injuries. Scarring from injuries, surgeries, and burns places a significant burden on the healthcare system. Patients with major scars, especially children and adolescents, often experience long-term functional and psychological issues. This article aims to present a case of post-surgical hypertrophic scar in a patient after gap arthroplasty through Al-Kayat Bramley incision and the role of a multi-professional team to treat such wounds.

## Introduction

Wound healing is a natural phenomenon involving a complex interplay between cell types, cytokines, chemical mediators, and the vascular system. This process is essential for survival and involves a well-orchestrated sequence of events, including hemostasis, inflammation, proliferation, and remodeling [1]. The process of wound healing begins with hemostasis, which occurs immediately after injury. The primary goal during this phase is to stop bleeding and prevent further blood loss by vasoconstriction and platelet aggregation to form a temporary hemostatic plug. This is followed by coagulation cascade activation and clot formation which serves as a scaffold for incoming cells crucial for the next stages of healing. Inflammation is the second phase where the recruitment of immune cells such as neutrophils and macrophages occurs. Neutrophils perform the phagocytic activity and clear the debris allowing for decontamination of wounds and macrophages secrete cytokines and growth factors which orchestrate the next phase by attracting cells essential for tissue formation and repair [2]. The proliferation phase is marked by the formation of new tissue and includes angiogenesis, fibroplasia, and re-epithelialization. The final phase of wound healing is remodeling which can last for months to years. During this phase, the newly formed tissue is reorganized and strengthened, following which collagen fibers are realigned, and excess cells are removed through apoptosis. The tensile strength of the wound increases as collagen becomes more cross-linked and organized. In their morpho-elastic model for dermal wound closure, Bowden et al. concluded that the ultimate resulting wound will never have 100% of its original strength and only about 80% of the tensile strength [3].

Pathological conditions such as temporomandibular joint (TMJ) ankylosis, a bony or fibrous adhesion of joint components such as the mandibular condyle and the glenoid fossa of the temporal bone, always require surgery as the first line of treatment [4]. As extraoral surgical approaches are mandatory for gaining access to the TMJ region, chances of scarring post-surgery is an unavoidable finding. In their study involving 150 patients on postoperative follow-up evaluating nerve function, hemorrhage, and esthetic outcomes of the preauricular approach to the TMJ, Nellestam and Eriksson concluded that none of the patients considered the resultant scar disfiguring [5]. Furthermore, there is no documented evidence of hypertrophic scar formation following gap arthroplasty performed using the Al-Kayat Bramley incision.

## Case presentation

**Figure 1:**
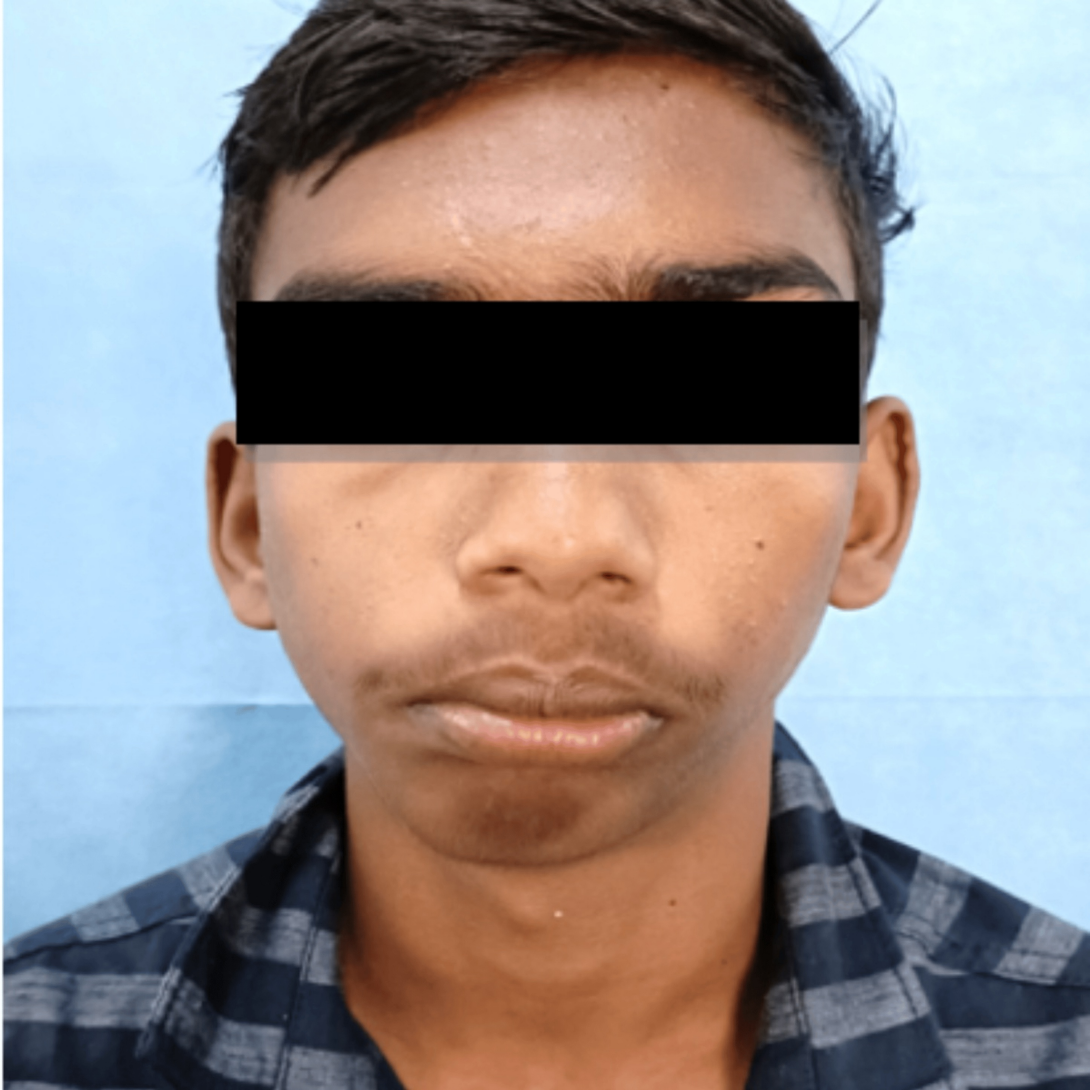
Preoperative clinical photograph: frontal view. The preoperative frontal view of the patient showing facial asymmetry, fullness of the face on the right side, flattening of the face on the left side, and deviation of the chin toward the right side.

**Figure 2:**
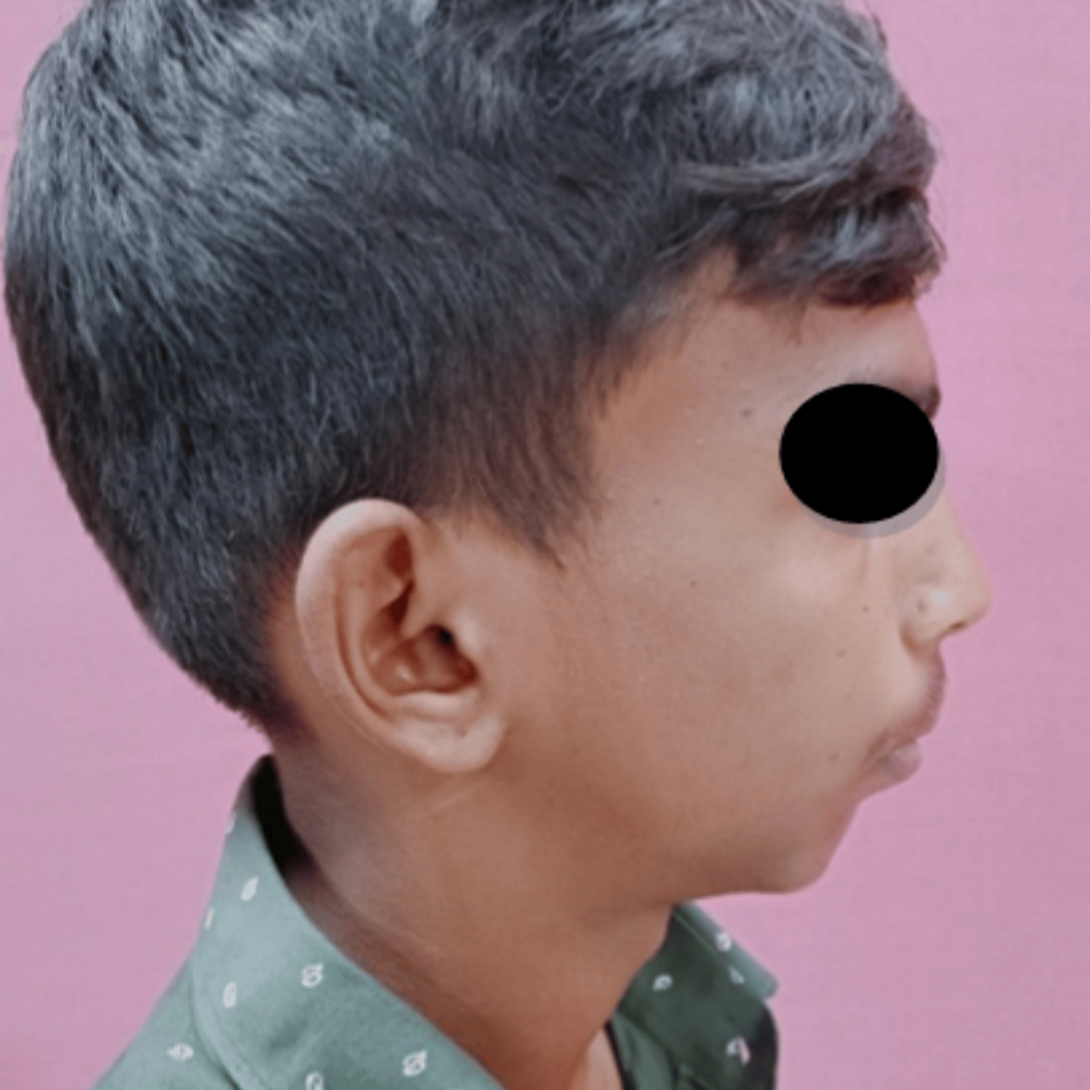
Preoperative clinical photograph: profile view (right side). Profile view of the patient showing a convex facial profile and retrognathic mandible.

**Figure 3:**
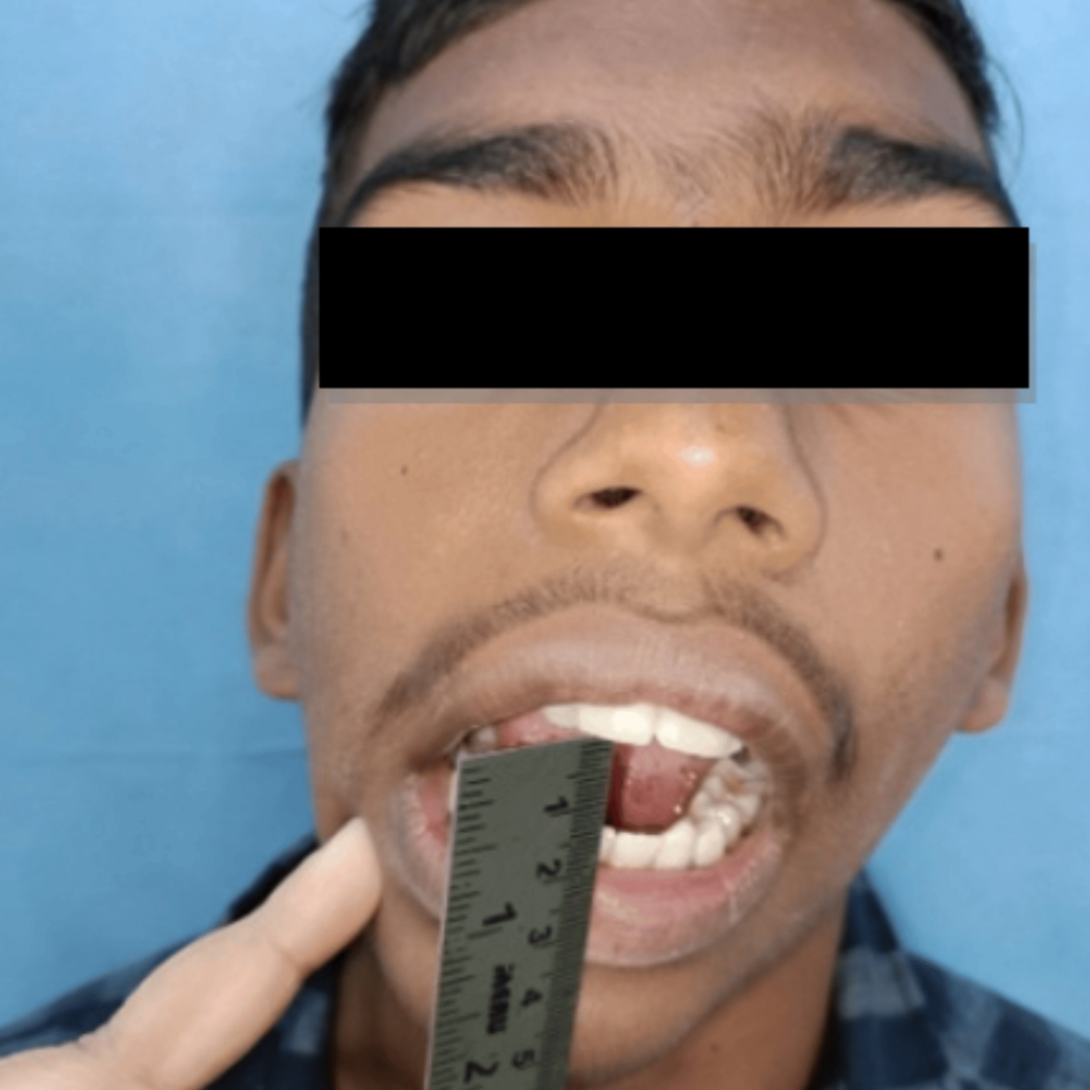
Preoperative mouth opening measuring 12 mm.

A 16-year-old male patient reported to the Department of Oral and Maxillofacial Surgery with a complaint of restricted mouth opening since childhood. A history of trauma during childhood was recorded. On clinical examination, the patient had gross facial asymmetry, a convex facial profile, fullness of face on the right side, flattening of the face on the left side, restricted mouth opening of approximately 12 mm, and a deviation of the mandible toward the right side on opening and closing the mouth (Figures 1-3).

**Figure 4:**
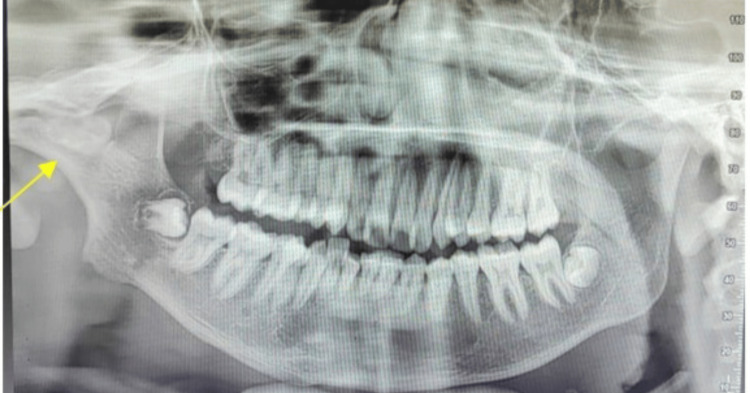
Preoperative orthopantomogram showing right-sided TMJ ankylosis.

**Figure 5:**
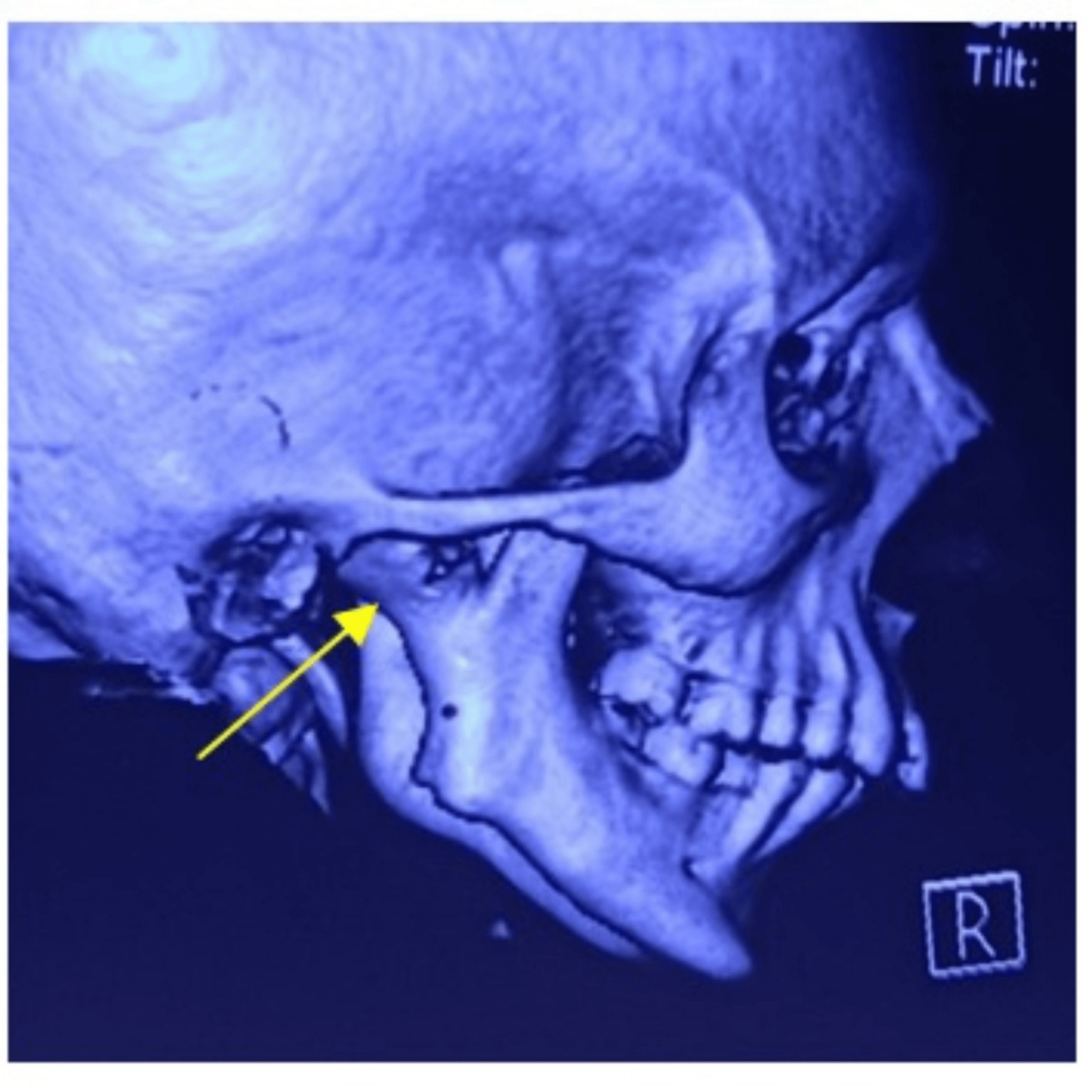
CT of the facial bones showing right-side temporomandibular joint ankylosis.

**Figure 6:**
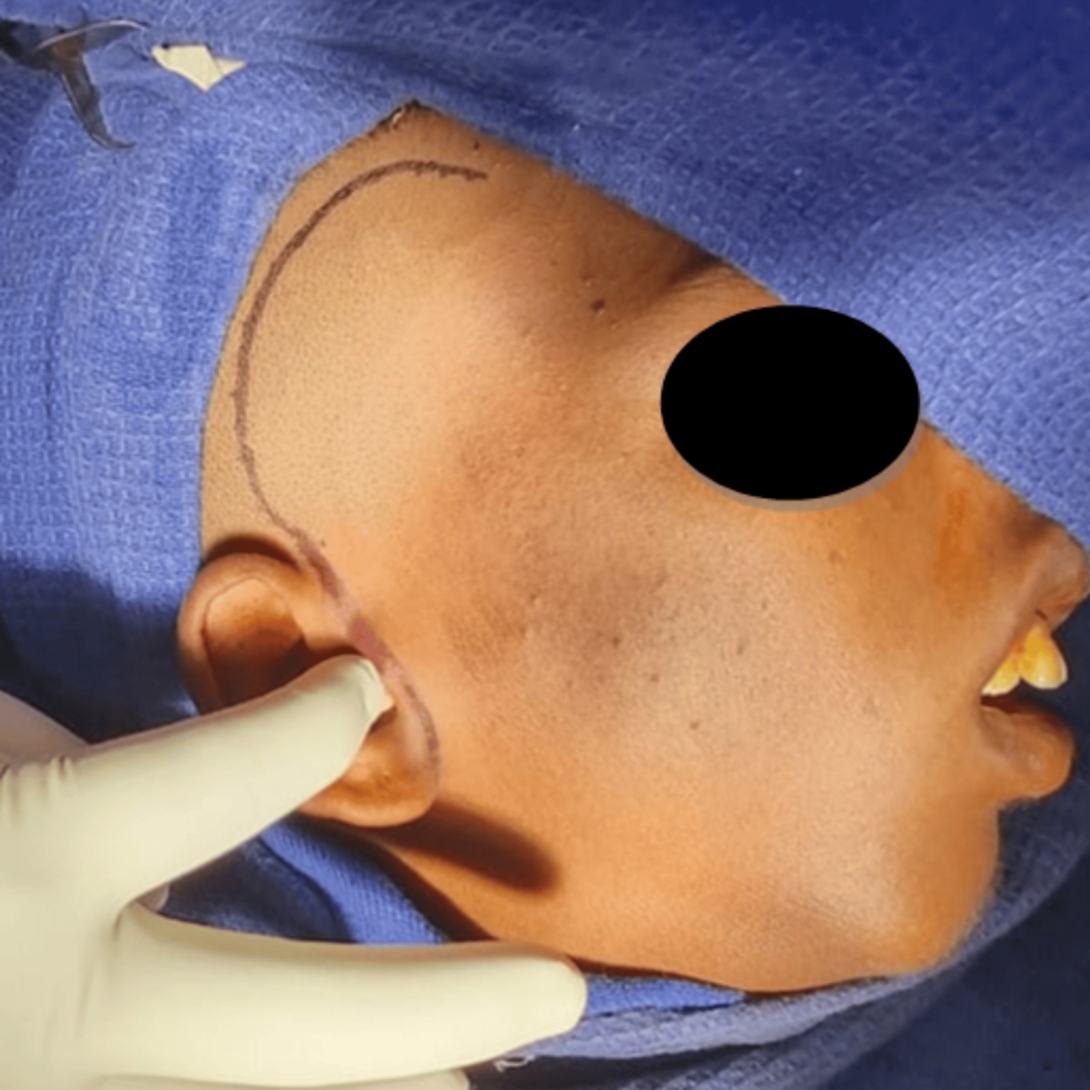
Marking of the Al-Kayat Bramley incision.

Orthopantomogram (OPG) and CT of the facial bones revealed a fusion of the right side of the mandibular condyle to the glenoid fossa of the temporal bone suggesting unilateral TMJ ankylosis (Figures 4, 5). Gap arthroplasty was performed under general anesthesia through the Al-Kayat Bramley incision (Figure 6).

Surgical procedure

**Figure 7:**
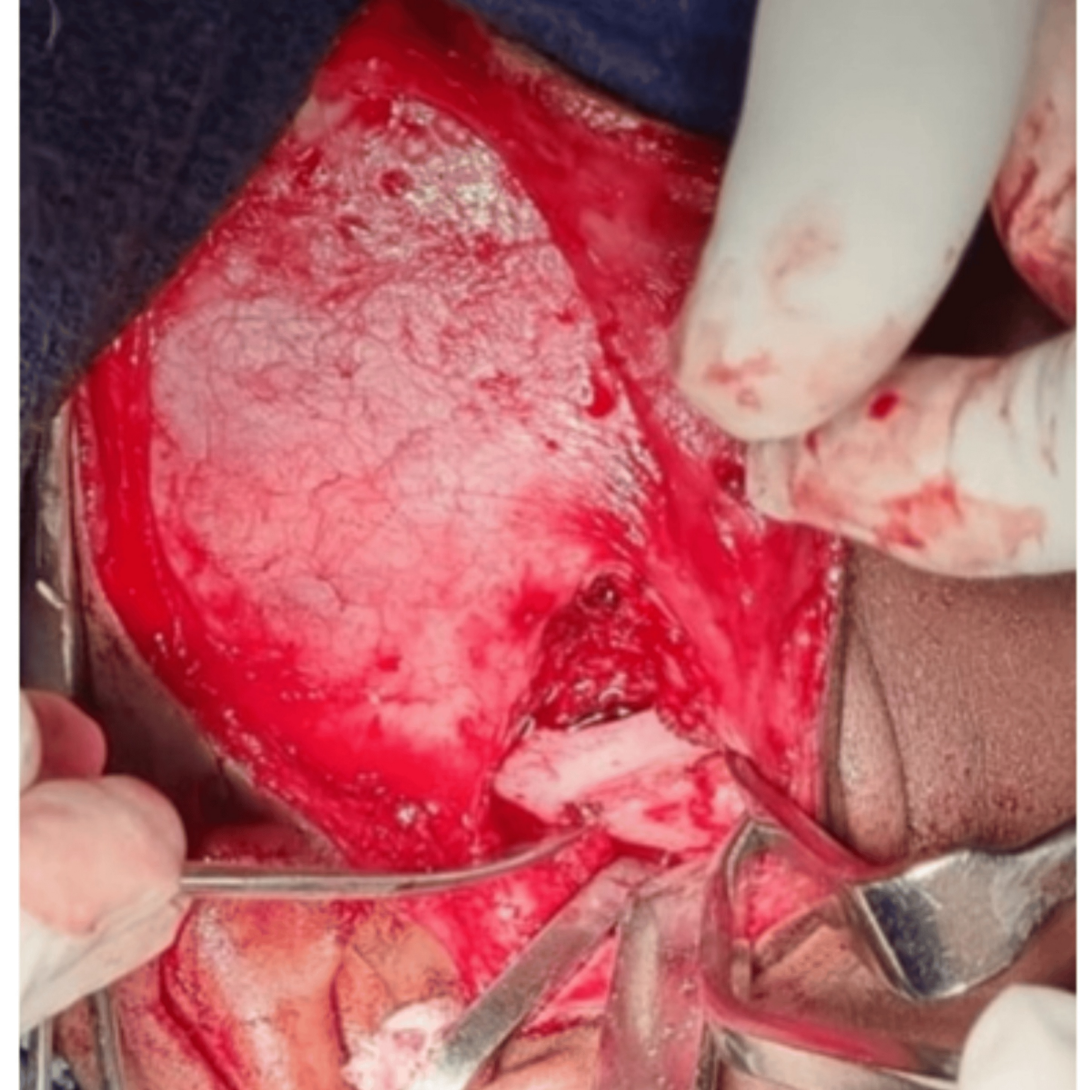
Ankylotic mass exposed over the right temporomandibular joint region.

Under awake fiberoptic intubation, general anesthesia was induced and maintained. An Al-Kayat Bramley incision was placed from within the hairline and extended inferiorly until the root of the tragus. Layerwise, dissection was done and the ankylotic mass was exposed. After determining the anterior and posterior limits of the ankylotic mass, the segment was resected using burs, osteotomes, and mallet, creating a 15-20 mm gap (as per Kaban’s protocol) between the bony stumps (Figure 7).

**Figure 8:**
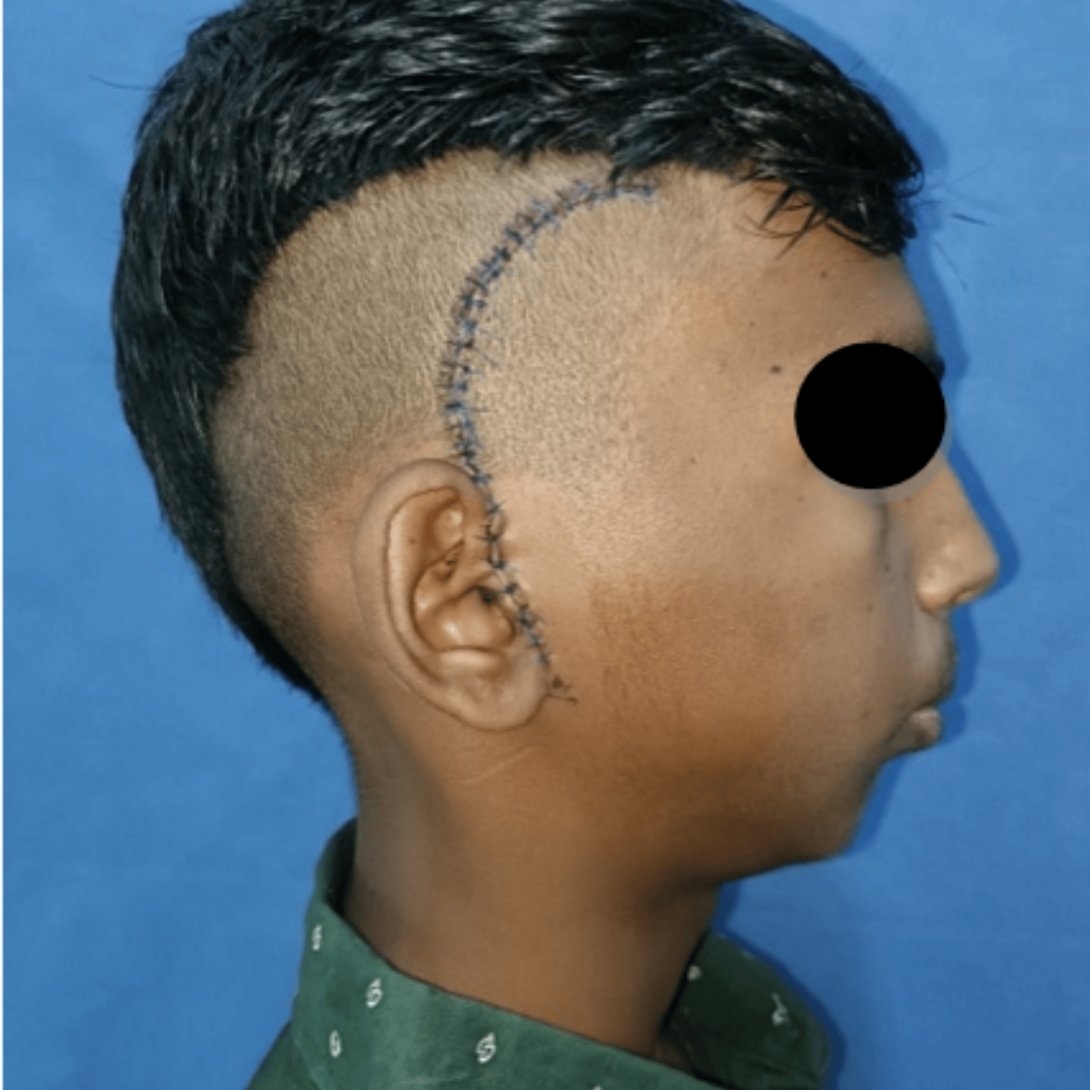
Surgical wound five days post-surgery.

**Figure 9:**
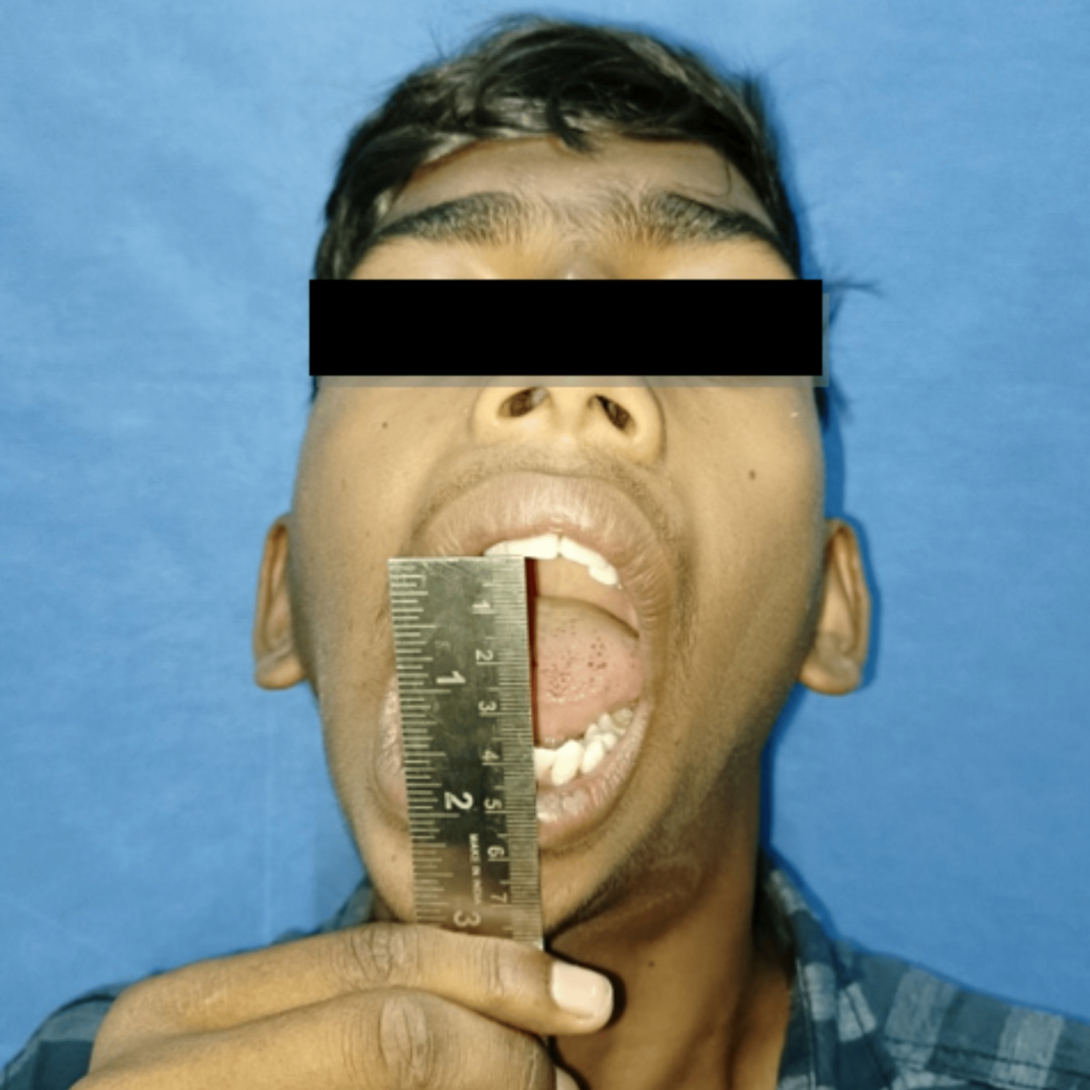
Postoperative mouth opening measuring 40 mm.

**Figure 10:**
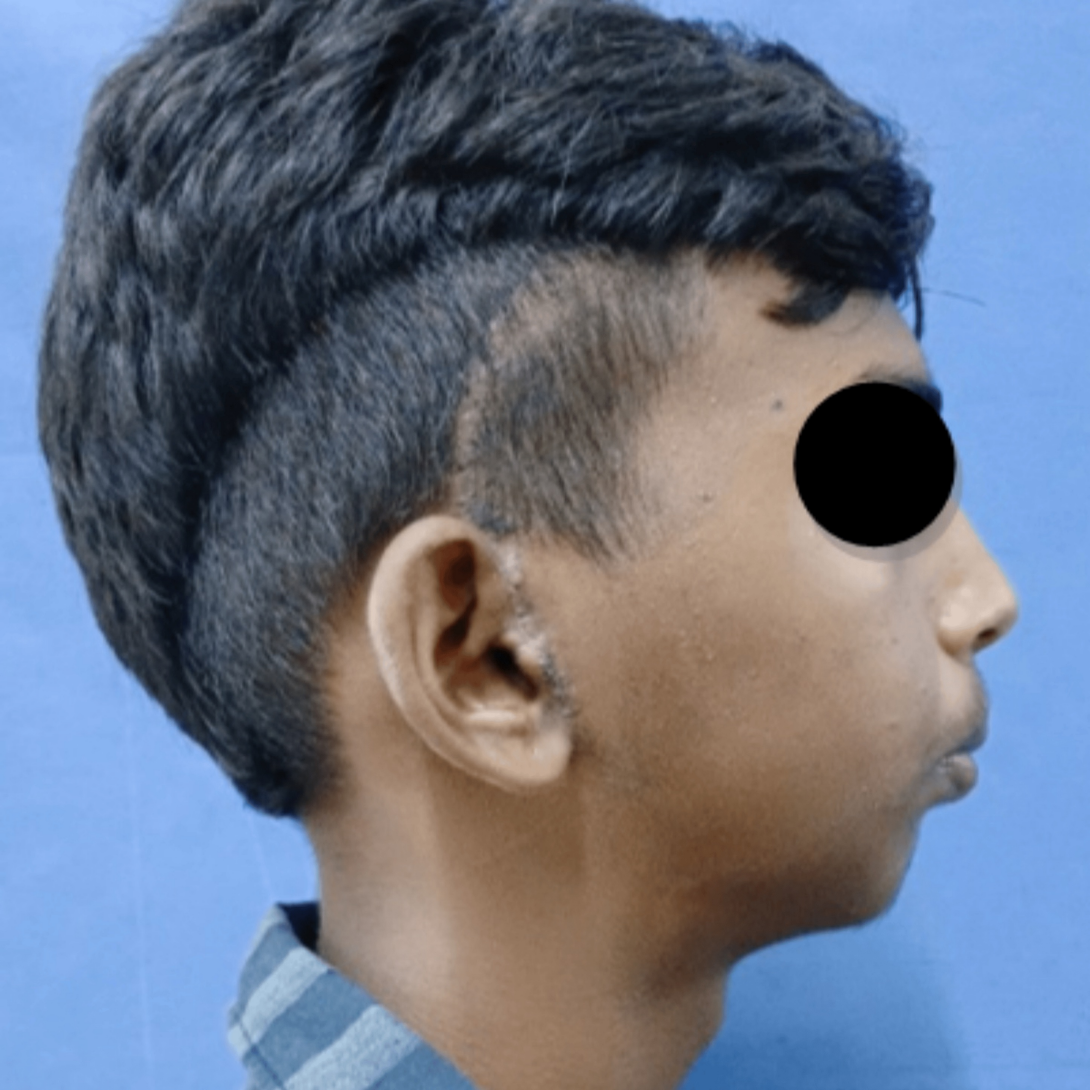
Surgical wound one month post-surgery. The surgical wound is healthy in the first month post-surgery with no signs of pathological scarring.

A mouth opening of 40 mm was achieved. Layerwise, closure was done using 3-0 polyglactin 910, and the skin layer was closed using 4-0 polypropylene suture. A pressure dressing was placed and removed after two days. Postoperatively, intravenous antibiotics and analgesics were administered for five days. The surgical wound was healthy with no signs of secondary infection during the hospital stay (Figure 8). The patient was advised to perform active mouth-opening exercises. His mouth opening after surgery was 40 mm (Figure 9). The patient’s surgical wound was healthy in the first month after surgery (Figure 10).

**Figure 11:**
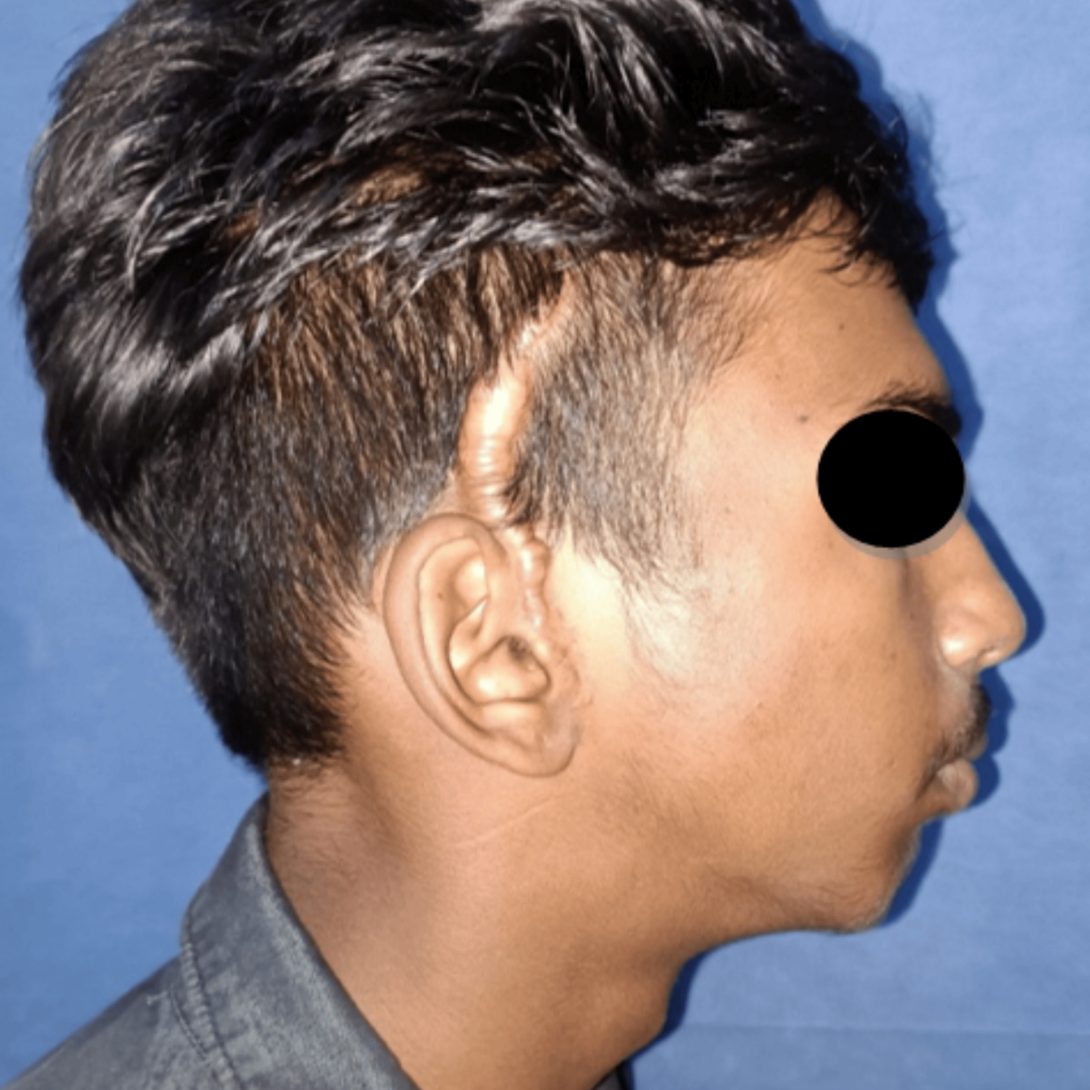
Hypertrophic scar during 11 months post-surgery. Pathological scarring seen along the incision site at 11 months post-surgery.

**Figure 12:**
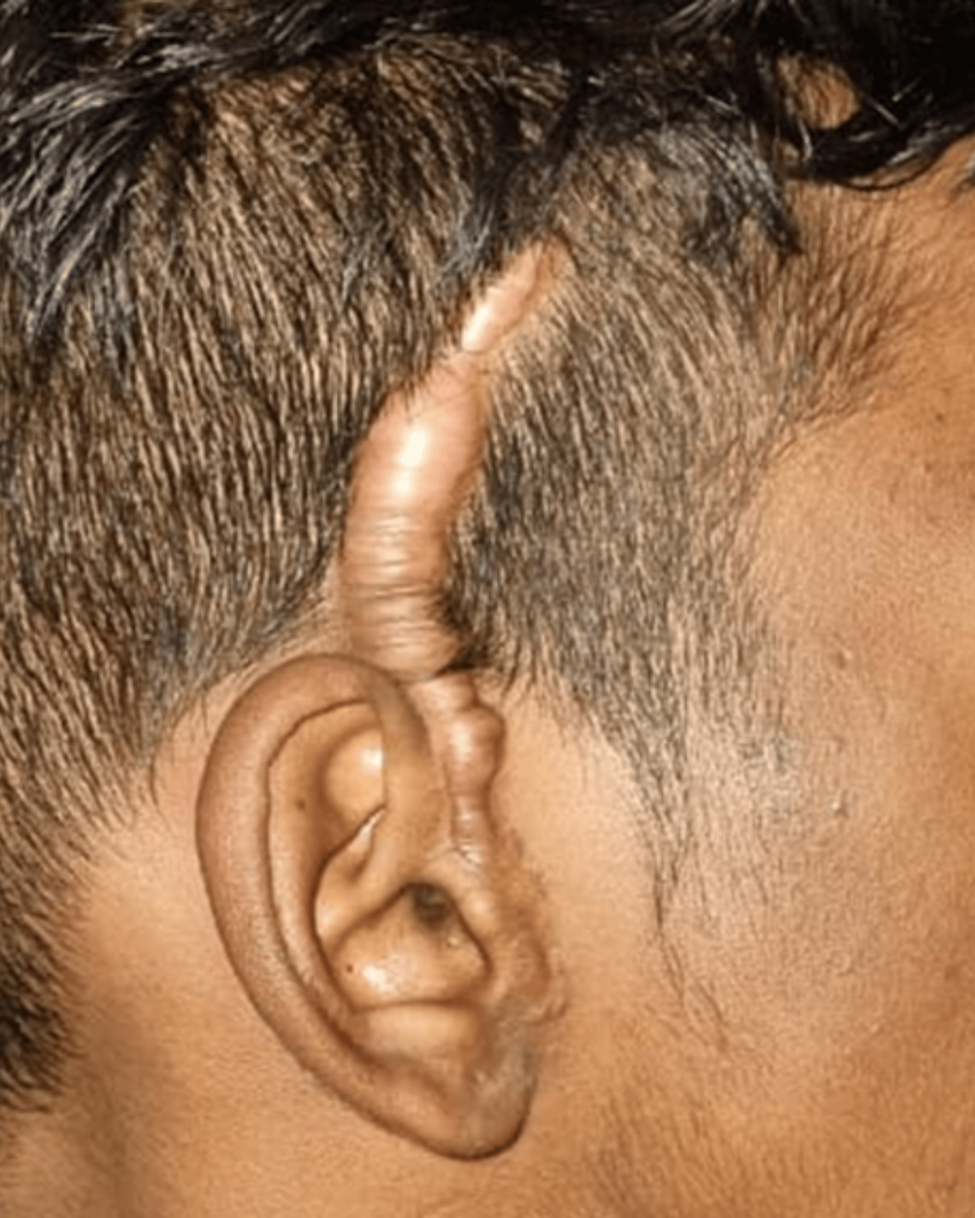
Hypertrophic scar over the incision site.

Seven months post-surgery, the patient reported a hypertrophic scar over the surgical incision site with alopecia on the affected site which was initially small but gradually increased in size to approximately 8 × 1 cm and was associated with mild pruritis (Figures 11, 12).

**Figure 13:**
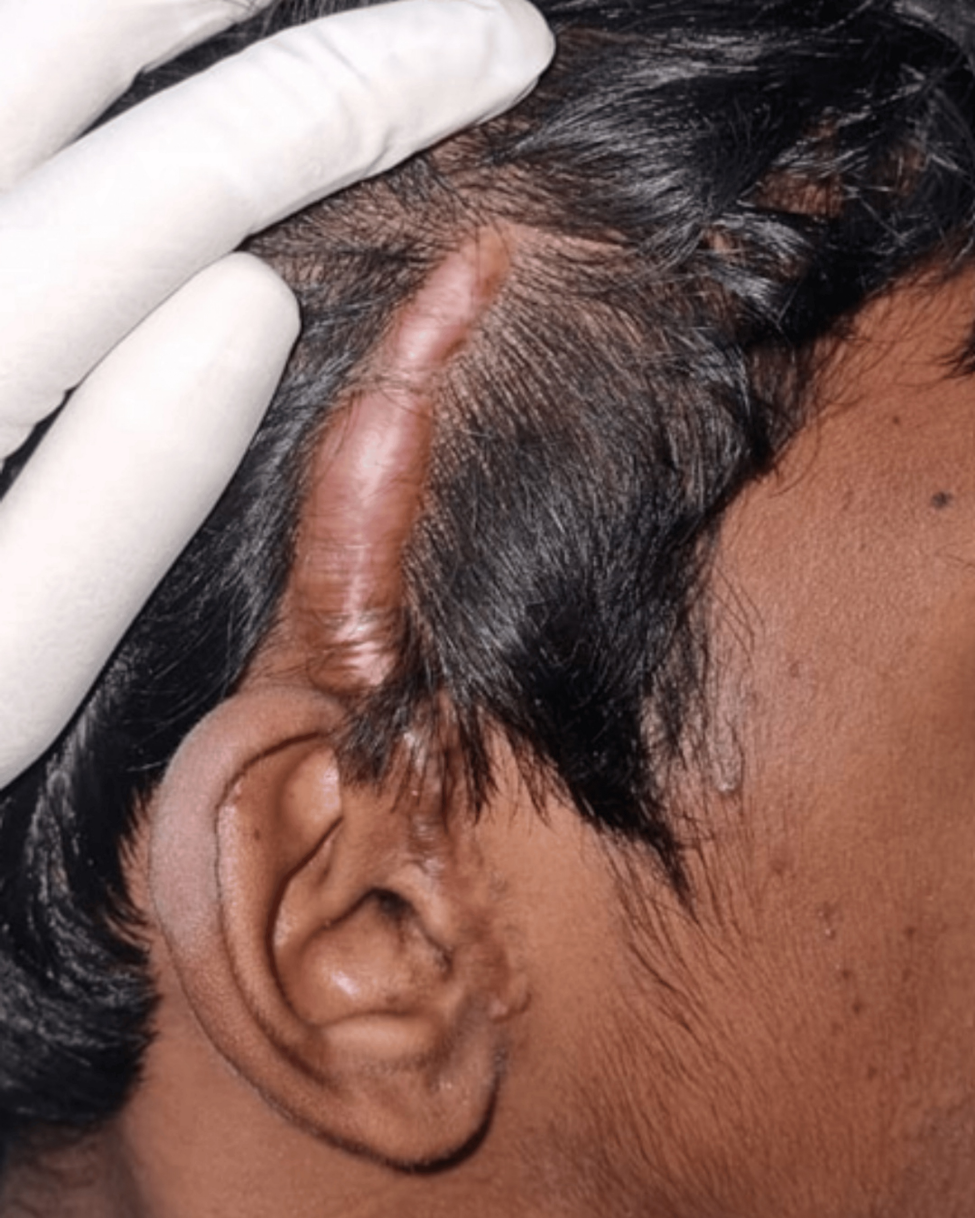
Scar after three sessions of cryotherapy. The scar has reduced in size after three sessions of cryotherapy.

No evidence of infection was noted. A dermatologist’s opinion was obtained. Topical steroids including 0.1 % betamethasone ointment for local application were given and further cryotherapy with liquid nitrogen was planned by the dermatologist. There was a reduction in the size of the scar after the third session of cryotherapy (Figure 13). Following cryotherapy, the patient was treated with intralesional steroids.

## Discussion

Disruptions in cutaneous continuity change the cellular mechanical environment and can impact the cells involved in the wound-healing cascade. Once cutaneous integrity is restored, mechanical forces may guide skin remodeling, resulting in a strong and lasting repair. The mechanisms through which these processes occur in normal skin and during wound healing remain unclear. Wound healing is divided into the following three stages: inflammation, proliferation, and remodeling. The inflammation stage starts with the disruption of capillary blood vessels and the formation of a fibrin clot. The proliferation stage begins around day four or five, marked by fibroblasts migrating into the wound matrix. By two weeks, these fibroblasts produce collagen fibers, firming up the wound. The final stage, remodeling, starts approximately three weeks post-injury, characterized by a reduction in fibroblast numbers, occlusion of blood vessels, and hardening of collagen fibers, leading to the firmness and reduced elasticity of scars. Continuous collagen production and degradation influence the remodeling of the mature wound matrix for approximately six months after an injury. At this stage, the production and breakdown of collagen reach equilibrium, resulting in no significant change in collagen levels. This remodeling phase is critical in determining variations in scar quality between different individuals. During this period, a healing incisional wound can potentially develop into an unsightly scar. Changes in the wound’s microenvironment, including shifts in mechanical forces, oxygen levels, chemokine activity, extracellular matrix composition, and growth factor production, directly influence cellular recruitment and activation, potentially causing impaired wound healing. Abnormal wound repair can manifest as either excessive fibrosis and scarring or insufficient healing, as in chronic non-healing ulcers. Both extremes of wound healing dysfunction present significant healthcare challenges.

A scar is the body’s natural way of healing to replace lost or damaged tissues. Scar formation is a normal physiological response to wounding in adults. However, when there is an alteration of extracellular matrix metabolism, resulting in an imbalance between its breakdown and buildup, it can lead to excessive scarring [6]. A hypertrophic scar can be defined as a scar forming after an injury that is larger or more raised than usual or that results in contracture. A hypertrophic scar is formed as a result of an increased number of fibroblasts and excessive type III collagen production [7]. They represent undesirable variants in the wound-healing process. Hypertrophic scars can be caused by both genetic and non-genetic factors such as smoking, infection, site of the wound, and suture material used. Genetic factors include chromosomal alterations in 2q23, 7p11, and 10q23.31 [8]. A hypertrophic scar often develops on the areas of the body that experience the most skin tension such as the back, chest, upper arms, and skin over a bony prominence [9]. Recent evidence has shown that the skin and its constituent cells are highly sensitive to mechanical stimuli, and that dysregulated mechano-transduction contributes to various wound-healing disorders. Mechano-transduction pathways, particularly the integrin-FAK pathway, play a crucial role in these processes. FAK activation in fibroblasts leads to increased hypertrophic scar formation, while reduced FAK activity is linked to chronic non-healing wounds, highlighting its importance in cutaneous repair [10]. A hypertrophic scar is more likely to occur in burn wounds and cases of infected wounds. It can occur in all age groups and affects both the physical and psychological well-being of the patients. Physical problems include pruritis, pain, and stiffness. Overexpression of transforming growth factor-beta 1 (TGF-β1) leads to increased stimulation of fibroblasts which results in increased elastin and collagen deposition in the wound. Fibroblasts isolated from hypertrophic scar express higher levels of TGF-β1 and TFG-β receptors, suggesting that an exaggerated TGF-β feedback loop may contribute to the hypertrophic scar phenotype [11]. Increased collagen deposition raises the wound tension which further predisposes to scarring. Hypertrophic scars often remain within the boundaries of the original wound and are characterized by induration and depigmentation. Diagnosis is usually made based on clinical findings. If the scarring worsens or continues to change biopsy may be needed. Reducing tension on the wound can prevent hypertrophic scar formation in surgical wounds. Hypertrophic scars can be distinguished from keloids in that hypertrophic scars remain within the borders of the original wound while keloids grow beyond the borders. Hypertrophic scars are associated with contractures and regress spontaneously while keloids do not [12].

Patients who are prone to developing hypertrophic scars should avoid elective surgeries. However, as TMJ ankylosis is a deteriorating condition, surgery is unavoidable. Hypertrophic scar over the Al-Kayat Bramley incision site is a rare finding.

The phases of wound healing overlap and are not distinctly separate. The proliferation phase starts before the inflammation phase has fully resolved and continues into the remodeling phase. The remodeling phase extends well beyond the removal of sutures and the removal of dressings. Therefore, incisional wound care should be viewed as a continuous process, with long-term goals focused on minimizing scar. Emphasizing these aspects of wound management is crucial to encourage patient involvement. It seems more efficient to prevent excessive scars than to treat them. Historically, the most recommended strategy has involved prophylactic measures such as silicone gel sheeting or paper tape starting two weeks post-injury, in conjunction with other treatments. For incisional scar management, modifiable factors include the design of the incision, gentle handling of soft tissue, ensuring hemostasis, maintaining aseptic techniques, and employing tension-reducing methods in the immediate and long-term postoperative periods [13]. In our patient, the first appearance of the hypertrophic scar was observed seven months after surgery, likely during the remodeling phase of wound healing.

Various modalities of treatment are available for hypertrophic scar such as conservative, medical, and surgical. The most successful treatment of a hypertrophic scar or keloid is achieved when the scar is immature but the overlying epithelium is intact. Conservative techniques such as occlusive dressings and compressive therapy work best immediately after surgery or injury. Occlusive dressings work by reducing the blood supply, oxygen, and nutrients to the scar, thereby reducing collagen synthesis. Compressive therapy such as ACE bandages, button compression, and spandex bandages apply local pressure to the affected area causing thinning of skin and reducing the cohesiveness of collagen fibers [14]. Medical management with steroids is a commonly used approach. Steroids, either topical application or intralesional, can be administered to reduce the production of pro-inflammatory mediators and collagen. Our patient is currently undergoing intralesional steroid therapy once every six weeks. Surgical removal of hypertrophic scar is performed to reduce the tension, often resulting in recurrence. Better results and lower recurrence are obtained if surgery is performed along with other modalities such as steroids. Cryotherapy is another available option that involves the application of a cold source to the scar, typically liquid nitrogen. The mechanism of action likely involves occlusion of the microcirculation, thereby inducing cell injury, causing necrosis of the affected area, and reducing the size of the scar. A semi-controlled study by Berman et al. showed that combined treatment with corticosteroid injection and cryotherapy was superior to either treatment alone. In our patient, a combination of intralesional steroids and cryotherapy with liquid nitrogen was performed, and after three sessions, the patient showed improvement in the scar. Cryotherapy works best for small scars with narrow bases allowing the concentration of the cooling effect and maximizing freezing [15].

## Conclusions

Scar formation, especially in the facial region, poses a significant challenge for surgeons. Genetic predisposition along with various other factors influence the nature and the frequency of scar formation. In patients with TMJ pathologies such as ankylosis, surgery being the mainstay treatment, scar formation is anticipated. Our experience has shown the development of pathological scars with the Al-Kayat Bramley incision. It is crucial to incorporate preventive measures in the treatment plan and educate the patients about the possibility of scar formation. Hypertrophic scarring on the face is rare, and oral and maxillofacial surgeons should be aware of such potential postoperative complications. Routine clinical practice should include taking the history of any tendency to develop pathological scars. Our case underlines the importance of regular follow-ups to monitor both the mouth opening and facial esthetics. Patients with TMJ ankylosis, particularly adolescents to teenage age groups, are often stigmatized. Hence, appropriate management of postoperative scarring is essential to maintain the patient’s physical, psychological, and social well-being.
